# Model Predictions of Occupational Exposures to Diacetyl and 2,3-Pentanedione Emitted From Roasted Whole Bean and Ground Coffee: Influence of Roast Level and Physical Form on Specific Emission Rates

**DOI:** 10.3389/fpubh.2022.786924

**Published:** 2022-03-23

**Authors:** Ryan F. LeBouf, Anand Ranpara, Elizabeth Fernandez, Dru A. Burns, Alyson R. Fortner

**Affiliations:** Respiratory Health Division, National Institute for Occupational Safety and Health, Morgantown, WV, United States

**Keywords:** diacetyl, 2, 3-pentanedione, coffee, emission rate, occupational exposures, volatile organic compounds

## Abstract

Roasted coffee emits hazardous volatile organic compounds including diacetyl and 2,3-pentanedione. Workers in non-flavored coffee roasting and packaging facilities might inhale diacetyl and 2,3-pentanedione from roasted coffee above occupational exposure limits depending on their work activities and proximity to the source of emissions. Objectives of this laboratory study were to: (1) investigate factors affecting specific emission rates (SERs) of diacetyl and 2,3-pentanedione from freshly roasted coffee, (2) explore the effect of time on SERs of coffee stored in sealed bags for 10-days, and (3) predict exposures to workers in hypothetical workplace scenarios. Two roast levels (light and dark) and three physical forms (whole bean, coarse ground, and fine ground) were investigated. Particle size for whole bean and ground coffee were analyzed using geometric mean of Feret diameter. Emitted chemicals were collected on thermal desorption tubes and quantified using mass spectrometry analysis. SERs developed here coupled with information from previous field surveys provided model input to estimate worker exposures during various activities using a probabilistic, near-field/far-field model. For freshly roasted coffee, mean SER of diacetyl and 2,3-pentantedione increased with decreasing particle size of the physical form (whole bean < coarse ground < fine ground) but was not consistent with roast levels. SERs from freshly roasted coffee increased with roast level for diacetyl but did not change for 2,3-pentanedione. Mean SERs were greatest for diacetyl at 3.60 mg kg^−1^ h^−1^ for dark, fine ground and for 2,3-pentanedione at 3.88 mg kg^−1^ h^−1^ for light, fine ground. For storage, SERs of whole bean remained constant while SERs of dark roast ground coffee decreased and light roast ground coffee increased. Modeling demonstrated that near-field exposures depend on proximity to the source, duration of exposure, and air velocities in the near-field further supporting previously reported chemical air measurements in coffee roasting and packaging facilities. Control of source emissions using local exhaust ventilation especially around grinding activities as well as modification of work practices could be used to reduce exposures in this workforce.

## Introduction

Between 2015 and 2017, the U.S. National Institute for Occupational Safety and Health (NIOSH) received 17 Health Hazard Evaluation requests at coffee roasting and packaging facilities and cafés. As a part of the requests, NIOSH researchers investigated personal exposures and area air concentrations of diacetyl, 2,3-pentanedione, and other volatile organic compounds (VOCs) (1). They found elevated worker exposures to diacetyl and 2,3-pentanedione when working around sources of ground roasted coffee and during grinding tasks (1). Roasted coffee emits VOCs, carbon monoxide, and carbon dioxide at various rates depending on the origin, processing, roast level, physical form, and storage conditions of the coffee (2). Researchers have observed an increase in specific emission rates (SERs) for carbon monoxide with increasing roast level (i.e., darker roasts) and with ground coffee compared to whole bean (3). These researchers also raised concern about storing roasted coffee in unventilated or under-ventilated storage areas because of carbon monoxide accumulation in the space to unsafe levels. The same concerns could be raised for the buildup of hazardous VOCs in storage bins and containers of roasted coffee or in under-ventilated storage areas. Diacetyl and 2,3-pentanedione have also been found in flavoring formulations used to impart a buttery smell or taste to baked goods and in electronic cigarette liquids (1, 4–8). Diacetyl exposure via inhalation has been associated with a debilitating lung disease, obliterative bronchiolitis (9). Like diacetyl, previous studies on animals demonstrated similar respiratory toxicity for 2,3-pentanedione (10).

*Coffea arabica* and *Coffea robusta* are the two species of coffee commonly used for roasting (11). Roasting green coffee beans at temperatures at or above 200°C (11, 12) produces a myriad of chemicals via the Maillard reaction, Strecker degradation, pyrolysis, and other chemical reactions that give roasted coffee a characteristic aroma (13). Over 800 compounds have been identified from roasted coffee (14). The constituents of coffee emissions include furans, pyrazines, pyrroles, sulfur compounds, aldehydes, and ketones including the alpha-dicarbonyl species: glyoxal, methylglyoxal, diacetyl, and 2,3-pentanedione (14–17). Average concentrations of chemicals in brewed coffee have been measured at 8 μg glyoxal/g, 152 μg methylglyoxal/g, and 19 μg diacetyl/g dry coffee (15). The type of coffee and origin can also affect relative concentration of chemicals formed. Using dynamic headspace analysis to characterize the volatile composition of roasted coffee, a greater concentration of diacetyl and 2,3-pentanedione was measured for Arabica samples (3,235–8,818 μg diacetyl/kg and 3,087–8,853 μg 2,3-pentanedione/kg) compared to Robusta samples (1,959–4,316 μg diacetyl/kg and 341.1–4,701 μg 2,3-pentanedione/kg) (18). Colzi et al. observed a similar trend of greater VOC emissions in terms of type and quantity from Arabica compared to Robusta when attempting to characterize and distinguish species based on volatile profiles using proton transfer time-of-flight mass spectrometry (19). Mayer et al. measured differences in volatile emissions from different varietals of the same species. One research group has found varying concentrations of diacetyl and 2,3-pentanedione in *C. arabica* from different origins (20). Hyong et al. observed espresso coffee samples made using *C. arabica* from Brazil and Ethiopa had a greater concentration of diacetyl than *C. robusta* from Vietnam and India, and the concentration of diacetyl increased with roast temperature and time (21).

The duration and temperature at which coffee is roasted (i.e., roast level) can influence the aroma profile. Roast level has been shown to change the concentration of diacetyl in coffee beans that have been roasted longer. Chemometric analysis coupled with proton transfer time-of-flight mass spectrometry has been used to distinguish organic from regular coffee and espresso from other roast levels (22). The formation of diacetyl begins later in the roasting duration, at a medium roast level (210°C for 14 min) with a peak diacetyl concentration of 2.28 ± 0.07 mg/100 g between 14 and 16 min (23). Chemical reaction pathways change as the roast process continues with early stage roasting generating diacetyl from sucrose (the intact sugar skeleton) followed by sugar fragments later in the roasting cycle (24). Roast level also influences the pore structure of the roasted coffee bean, which can affect mass transport phenomena of aromatic compounds into the surrounding air (25). Bean porosity is increased during roasting because of cell destruction and degradation of the intercellular structure (11).

The physical form of the roasted coffee such as whole bean or ground can affect the rate of chemical release because of increased surface area (26, 27). Migration of coffee volatiles to the bean surface is a relatively slow process and can be limited by accumulation of volatiles into the headspace of the packaging material. Grinding the roasted coffee beans releases trapped aroma compounds and increases the emission rate of chemicals from roasted coffee. Coffee aroma (i.e., chemical emission) decreases over time during storage leading to staleness, a sweet but unpleasant sensory quality of taste and smell (16). Researchers have found that coffee aroma can be maintained when stored ground for ~2 weeks at room temperature depending on storage conditions (27). The rate of chemical emissions and the storage duration can be used to estimate total mass emitted. This calculated mass value can be compared to a measured value of total content in the bean. SERs estimated from different roast levels and forms of roasted coffee can be used to predict air concentrations of hazardous chemicals based on mass-balance models.

In this study, we employed a near/far field model with a constant emission rate to demonstrate the cyclic, diurnal pattern of high peak exposures when working close to source of emissions (near field) followed by low exposures (far field) and how this profile influences the prediction of full-shift occupational exposures. In laboratory tests, we estimated SERs of diacetyl and 2,3-pentanedione based on degree of roast and physical form to better understand exposure assessments during field investigations in coffee facilities. The aims of this laboratory study were to investigate the effect of roast level and physical form on SERs of diacetyl and 2,3-pentanedione released from freshly roasted and stored coffee, predict air concentrations of these chemicals in hypothetical work environments, and estimate occupational exposures assuming task and job work patterns based on real-world observations and information obtained from coffee roasting and packaging facilities.

## Methods

### Coffee Roasting

For each batch, 0.11 kg of green *C. arabica* beans (Colombia La Guamera, Sagebush Unroasted, Chandler, Arizona) was roasted in a BEHMOR Gourmet Coffee Roaster (Incline Village, Nevada) with smoke suppression technology and preprogrammed roast profiles. The roaster has a rotating metal drum cage with heating elements in the rear of the chamber. The roaster was preheated for 1 min and 45 s prior to roasting. During roasting, two distinct stages occur that can be heard by a cracking sound: (1) first crack when steam is rapidly released and the bean expands, and (2) second crack when the bean darkens and structural changes continue to occur. For hard beans, the equipment manufacturer recommends an automatic roast profile setting, P2, which reduces the power to the elements to 25%. Roast levels were achieved by roasting on the P2 setting for ~11 min (30 s after first crack) for light roast and ~14 min (30 s after second crack) for dark roast. We use the relative terms light and dark roast levels indicating that light is lighter than dark roast, the latter of which could be classified as medium roast based on a previous report (23). Roasts were visually observed for desired and consistent coloring.

### Coffee Grinding and Particle Sizing

**Figure 1:**
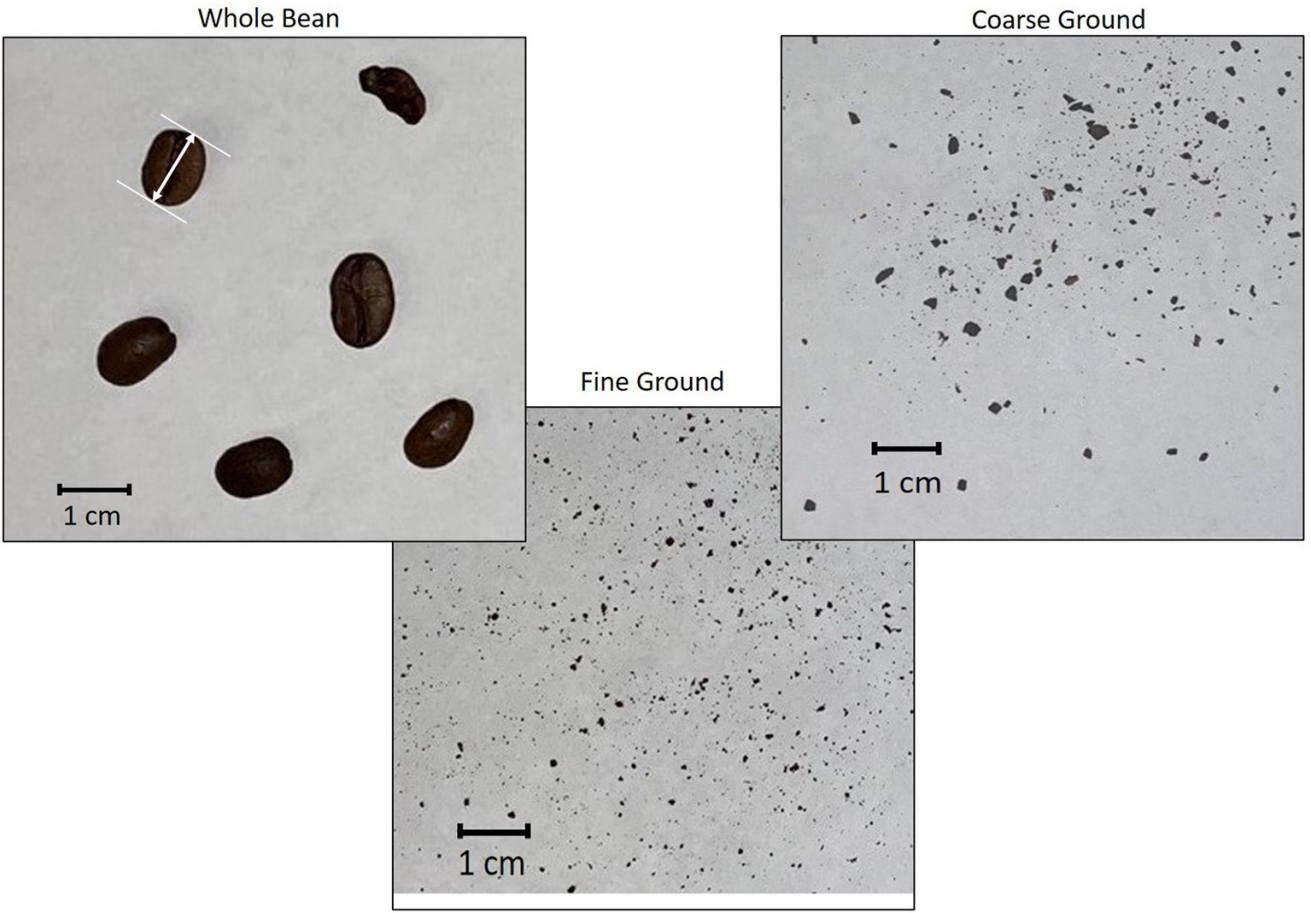
Photographs of the physical forms of dark roasted coffee (whole bean, coarse ground, fine ground). White arrow between two tangential lines is an example of Feret diameter.

The roasted beans were ground using a Cuisinart coffee grinder (DBM-8, Stamford, CT) on the coarsest or finest settings. Roasted coffee was assessed for particle size using ImageJ (public domain software produced at National Institutes of Health, Bethesda, MD). Whole bean or ground coffee was photographed on a piece of white paper with a ruler to set the scale in centimeters (Figure 1). Images were independently collected 3 times each for ground particles and 6 times for whole bean. Particles were sized using Feret or caliper diameter, which is the distance between two parallel tangential lines and indicates size along a specified direction.

### Experimental Design

Preliminary emission testing was conducted to determine appropriate roasting procedures and experimental set up. Chemical emission data was measured to assess the effect of emission factors of roast level and physical form on average SERs for two conditions: (1) freshly roasted and (2) stored coffee. Testing strategy for each factor depended on the conditions being tested (Table 1). For freshly roasted coffee, independently produced batches of coffee were roasted to include the variability associated with multiple roasts. For stored coffee, a single batch of each type of coffee was roasted to assess the impact of storage time on emissions. Storage emission samples were stored as whole bean, coarse ground, or fine ground. Storage emission samples were tested on approximately day 0 (within 4 h), 1, 4, and 10. Immediate roast emission samples were tested within 4 h of roasting and ground immediately before testing. One test was conducted for each freshly roasted sample while two tests were conducted for each stored coffee sample. Samples were stored in resealable, coffee storage bags with one-way valves on a shelf in the laboratory at ~22°C.

**Table 1:** Emission test conditions for freshly roasted and stored coffee.

		**Freshly roasted coffee**		**Stored coffee**	
		**Roast level**			
		**Dark**	**Light**	**Dark**	**Light**
Physical form	Whole bean	3 independent roasts (*n* = 3)	3 independent roasts (*n* = 3)	4 time points* 2 replicates per time point (*n* = 8)	3 time points** 2 replicates per time point (*n* = 6)
	Coarse ground	3 independent roasts (*n* = 3)	3 independent roasts (*n* = 3)	4 time points* 2 replicates per time point (*n* = 8)	3 time points** 2 replicates per time point (*n* = 6)
	Fine ground	3 independent roasts (*n* = 3)	3 independent roasts (*n* = 3)	4 time points* 2 replicates per time point (*n* = 8)	3 time points** 2 replicates per time point (*n* = 6)

* *4 time points: ~ Day 0 (within 4 hours), 1, 4, and 10*.

** *3 time points: ~ Day 0, 4, and 10. Storage emissions samples for light roast were not tested on day 1 because of scheduling conflicts*.

### Emission Sample Collection

The emissions test chamber (M-CTE 250; Markes International Inc., Sacramento, CA) was equilibrated for 20–30 min before each trial. Chamber temperature, flow rate, and relative humidity were measured before and after emission testing. Coffee emissions testing was performed using ultra-high purity air at 54.5 ± 11% relative humidity (RH; mean ± standard deviation) and 21.5 ± 2.5°C, measured with a Control Company 4,095 hygrometer/thermometer monitor (Webster, TX). Flow rate was controlled using an in-line rotameter and calibrated using a primary calibration flowmeter (Bios DryCal Defender 530, Mesa Laboratories, Butler, NJ) before and after testing and an average flow rate was used for emissions calculations. The air was humidified using a glass bottle containing 500 mL of water (18.2 MΩ-cm, Millipore Milli-Q system, Billerica, MA). To obtain the desired flow rate and temperature, the chamber system was operated in high-flow mode with the chamber heaters set to 25°C with the cooling fans on. The chamber exhaust air was sampled for diacetyl (2,3-butanedione, CAS# 431-03-8) and 2,3-pentanedione (CAS# 600-14-6) using Universal thermal desorption tubes (Part no. C3-CAXX-5266, inert-coated stainless steel, Markes International, Inc.) at a flow rate of 39.4 ± 2.2 mL/min. The test chamber had a volume of 114 mL, translating to ~20.7 air changes per hour (N, hr^−1^) at 39.4 mL/min.

Background air samples were collected from the test chamber for 20 min prior to placing coffee samples in each chamber to make sure the chamber air was clean and free of chemical interferents. Coffee samples were weighed to 5.2 ± 0.18 g (mean ± standard deviation) and placed into one of the four test chambers in the system. Some tests were conducted concurrently in two chambers operated in parallel. Eight sequential air samples were collected from each sample for both storage and roast emission tests at approximate midpoint times of 2, 4, 6, 8, 10, 15, 35, and 60 min. The first five time points were sampled for 30 s to capture rapid changes in chemical emissions. The last three time points were sampled for 1 min.

### Emission Sample Analysis

Samples were analyzed using a Markes ULTRA-XR, UNITY-XR thermal desorption system attached to an Agilent Technologies 6890 gas chromatograph/5977B mass spectrometer system. The thermal desorption parameters were as follows: internal standards bromochloromethane, chlorobenzene-d5 and 1,4-difluorobenzened added to the tube, split flow 50 mL min^−1^, flow path temperature of 150°C, desorption temperature of 280°C, purge time 1 min at 50 mL min^−1^, tube desorption time of 7 min with a flow of 50 mL min^−1^, and cold trap temperature of 25 up to 290°C during desorption. The gas chromatograph parameters were as follows: 2 mL min^−1^ helium flow, initial oven temperature 30°C (held for 5 min), temperature ramp of 5°C min^−1^ to 170°C, then 20°C min^−1^ to 220°C, with a final ramp of 33°C min^−1^ to 220°C. The mass spectrometer was operated in scan mode from 35 to 350 amu, mass spectrometer transfer line temperature 280°C, source temperature 300°C, and quadrupole temperature 150°C.

### Data Analysis

SER estimates were developed using chemical air concentration curves plotted against the midpoint of sampling duration. Curves were fitted using a non-linear regression technique, PROC NLIN, in SAS 9.4 (SAS Institute Inc., Cary, NC). SER models were based on these concentration curves and ASTM D5116 (28) modified with a steady state SER asymptote (EF_ss_). We used the first-order decaying source equation to fit the concentration curve data using Equation (1).


(1) $$C\left(t\right)\;=\;\left[\frac{L\left(EF_{0}\right)\left(e^{-kt}\;-\;e^{-Nt}\right)}{N\;-\;k}\right]\;+\;EF_{ss}$$

where C(t) = diacetyl or 2,3-pentanedione mass concentration, mg m^−3^, measured at midpoint time t,

L = loading factor (average value 45.6), which is the mass of material for each trial divided by the chamber volume, kg m^−3^,

EF_0_ = initial SER (0.1–5 by 1), mg kg^−1^ hr^−1^,

EF_ss_ = steady-state SER (0.1–5 by 1), mg kg^−1^ hr^−1^,

k = decay rate constant (0.1–4 by 1), hr^−1^,

N = air exchange rate, which is the flow rate of air for each trial divided by chamber volume of 114 mL, hr^−1^,

t = midpoint time, which is halfway between the start and end of the sample duration, hr.

Maximum predicted SERs for diacetyl or 2,3-pentanedione were calculated from the maximum concentration predicted that was chosen from the peak of the emission buildup and decay curve generated above. This peak corresponds to the time at which emission of chemical was equal to the removal. Maximum SERs were calculated using Equation (2).


(2) $$EF_{\text{max}}\;=\;C_{\text{max}}*\left(\frac{N}{L}\right)\;=\;C_{\text{max}}*\frac{Q}{m}$$

where EF_max_ = maximum SER, mg kg^−1^ hr^−1^,

C_max_ = maximum predicted diacetyl or 2,3-pentanedione concentration, mg m^−3^, from the fitted curve,

Q = volumetric flow rate, m^3^ hr^−1^,

m = mass of coffee, kg.

Air exchange rate (N) divided by the loading factor (L) can be reduced to the simpler form of Equation (2) that uses volumetric flow rate (Q) and mass (m). In the beginning of the trial, emission of chemical is greater than removal. The rate of accumulation during the buildup portion of the concentration curve will determine the adjusted maximum EF (EF_buildup_), which may be slightly higher than EF_max_. EF_buildup_ (mean ± standard deviation) was used for all data analysis. EF_buildup_ was calculated from EF_max_ using the following equation:


(3) $$\begin{array}{r} EF_{buildup}\;=\;\frac{EF_{\max}}{\left(1\;-\;e^{-Nt_{\max}}\right)} \end{array}$$

where EF_buildup_ = adjusted maximum emission factor accounting for buildup, mg kg^−1^ hr^−1^,

EF_max_ = maximum emission factor, mg kg^−1^ hr^−1^,

N = air exchange rate, hr^−1^,

t_max_ = time required to reach maximum predicted chemical concentration, hr.

Particle sizes (Feret diameter) of different forms of coffee (252 whole beans, 7,500 coarse ground particles, 10,819 fine ground particles) are summarized as geometric mean (GM) and geometric standard deviation (GSD) as these particle size distributions are log-normally distributed. Group-wise mean comparison tests on particle sizes for different forms of coffee were conducted on log-transformed data. SERs are summarized as average and standard deviation as these metrics are normally distributed. Minimum and maximum values are also presented. The effect of roast level, physical form, and the interaction between the two on SERs were investigated using a least-squares regression model with Student's *t*-test or Tukey's multiple comparison tests for groups of three or more at a significance level of 0.05 in JMP 13.0 (SAS Institute Inc., Cary, NC).

Emission factors are scalable quantities that can be converted to chemical generations rates based on the mass of material available for emission. These generation rates can then be used to estimate chemical air concentrations in a facility based on room volume and ventilation rates. We estimated chemical air concentrations using emission factors developed here and facility information from the Health Hazard Evaluations as model input.

We used a two-zone well-mixed box model with a constant emission source and IHMOD 2.0 (AIHA, Falls Church, VA, version 2.002, August 2018), a publicly available software, to calculate air concentrations. The near-field and far-field model equations can be found in Supplementary Material.

Model assumptions include instantaneously well-mixed concentration within each zone, air flow is limited between zones, cross-drafts (e.g., fans or equipment exhaust) are insignificant, initial chemical concentration in each zone is zero, chemical concentration of the supply air is zero, and the only removal of chemical from the zone is through exhaust (i.e., no losses to surfaces or chemical reactions). Total mass emitted in a certain time can be calculated to compare against known chemical content of material as a *post-hoc* assessment of the model to make sure the model is realistic and not overestimating contaminant transport from the material to the air. We assume that the chemical concentration of the air initially in the zones and entering the zones is zero.

Model inputs not described above in the equations were measured during Health Hazard Evaluation investigations, estimated from information observed during these investigations, or calculated based on laboratory-derived SERs reported here and assumed masses of coffee (Table 2). These scenarios represent realistic hypothetical workplace task durations, material quantities, production facility room volume, and ventilation (supply air) rates. Observations during field investigations indicated variable task durations and frequencies, but a cyclic work pattern of alternating proximity to the source was chosen for simplicity. For scenario A, we used air change rates based on measured values (supply air ventilation rates and room area) from a single facility during the Health Hazard Evaluation field investigations. Scenario A values are indicative of a small-scale coffee roasting and packaging facilities. The production room volume and material quantities for Scenario A are smaller than those in Scenario B, a hypothetical medium-scale coffee roasting and packaging facility.

**Table 2:** Input variables and values for model scenarios.

**Input variable**	**Scenario A values**	**Scenario B values**	**Scenario A information**	**Scenario B information**
Production room volume* (m^3^)	7,787	31,856	Measured/assumed; fixed value	Same information as A
Supply air (Q, m^3^ min^−1^)	65.9–80.5	238.9–292.0^†^	Measured 73.2 m^3^ min^−1^; uniform distribution with assumed 10% measurement error	Assumed; uniform distribution with assumed 10% measurement error
Generation rate^‡^ (G, mg min^−1^), dark roast, fine ground	0.6 ± 0.14	10.9 ± 2.6	Measured/assumed; ±95% confidence interval; normal distribution	Same information as A
Generation rate^‡^ (G, mg min^−1^), dark roast, whole bean	0.035 ± 0.24	0.64 ± 0.15	Measured/assumed, ±95% confidence interval; normal distribution	Same information as A
Mass of coffee used for each grinding or packaging task (kg)	10	181.8	Assumed based on small-production volume facility; fixed value	Assumed based on medium-production volume facility with large grinder capacity; fixed value

* *Assumed 7.6 m height; production room area measured in the field*.

† *Supply air equivalent to 0.5 air changes per hour used in scenario A, which was based on measured values in the field*.

‡ *Emission rate measured in laboratory tests; mass of coffee assumed*.

## Results

### Particle Sizing

The geometric mean particle sizes for different forms of coffee were 1.1 cm (GSD 1.5) for whole bean, 0.036 cm (GSD 2.0) for coarse ground, and 0.032 cm (GSD 1.9) for fine ground coffee (Table 3). A statistical difference was observed between whole bean and coarse or fine ground coffee (*p* <0.001) but no statistical difference between coarse and fine ground coffee (*p* = 0.17). Minimum and maximum particle size was larger for coarse (0.012; 0.9 cm) than fine ground coffee (0.008; 0.26 cm).

**Table 3:** Particle size measured as Feret diameter (cm) for whole bean, coarse ground, and fine ground forms of roasted coffee.

	**Feret diameter (cm)**			
**Physical form**	**Geometric mean**	**Geometric standard deviation**	**Minimum**	**Maximum**
Whole bean	1.1	1.5	0.80	1.38
Coarse ground	0.036	2.0	0.012	0.90
Fine ground	0.032	1.9	0.008	0.26

### Roast Level and Physical Form Effect on SERs

Mean SERs for diacetyl and 2,3-pentanedione increased with decreasing particle size of coffee form (whole bean < < coarse ground < fine ground) (Table 4). Whereas, the trend in mean SERs considering roast level (light, dark) was not consistent evidenced by SERs increasing for diacetyl but decreasing for 2,3-pentanedione as the roast level darkened. Mean SERs were greatest for diacetyl at 3.60 mg kg^−1^ h^−1^ for dark, fine ground and for 2,3-pentanedione at 3.88 mg kg^−1^ h^−1^ for light, fine ground. Variability measured as the coefficient of variation was greatest for light roast regardless of chemical when comparing between roast levels of the same ground form. Linear regression modeling for diacetyl revealed a significant effect of physical form (*p* <0.0001) and of roast level (*p* = 0.0067), while the interaction of form and roast level was not significant (*p* = 0.15). Linear regression modeling for 2,3-pentanedione revealed a significant effect of form (*p* <0.0001) but not for roast level (*p* = 0.82) nor the interaction of form and roast level (*p* = 0.80). Mean emission rates for physical forms were all significantly different from each other and the same was observed for roast levels, except for roast level comparison for 2,3-pentanedione (*p* = 0.82).

**Table 4:** Specific emission rates (*n* = 3) for diacetyl and 2,3-pentanedione for different roast levels (light, dark) and forms including whole bean, coarse ground, and fine ground.

			**Specific emission rates (mg kg^−1^ h^−1^)**				
**Compound**	**Roast level**	**Physical form**	**Mean**	**Std. dev**	**CV**	**Minimum**	**Maximum**
Diacetyl	Dark	Whole bean	0.21	0.14	67.9	0.09	0.37
		Coarse ground	2.50	0.32	12.8	2.22	2.85
		Fine ground	3.60	0.52	14.3	3.10	4.13
	Light	Whole bean	0.12	0.06	54.7	0.04	0.17
		Coarse ground	1.57	0.69	44.3	0.88	2.27
		Fine ground	2.34	0.77	33.0	1.46	2.92
2,3-Pentanedione	Dark	Whole bean	0.14	0.07	50.7	0.07	0.22
		Coarse ground	2.45	0.43	17.6	2.04	2.90
		Fine ground	3.43	0.58	17.0	2.77	3.88
	Light	Whole bean	0.07	0.05	77.9	0.02	0.12
		Coarse ground	2.33	1.17	50.3	1.34	3.63
		Fine ground	3.88	1.44	37.0	2.59	5.44

### Storage Duration Effect on SERs

**Figure 2:**
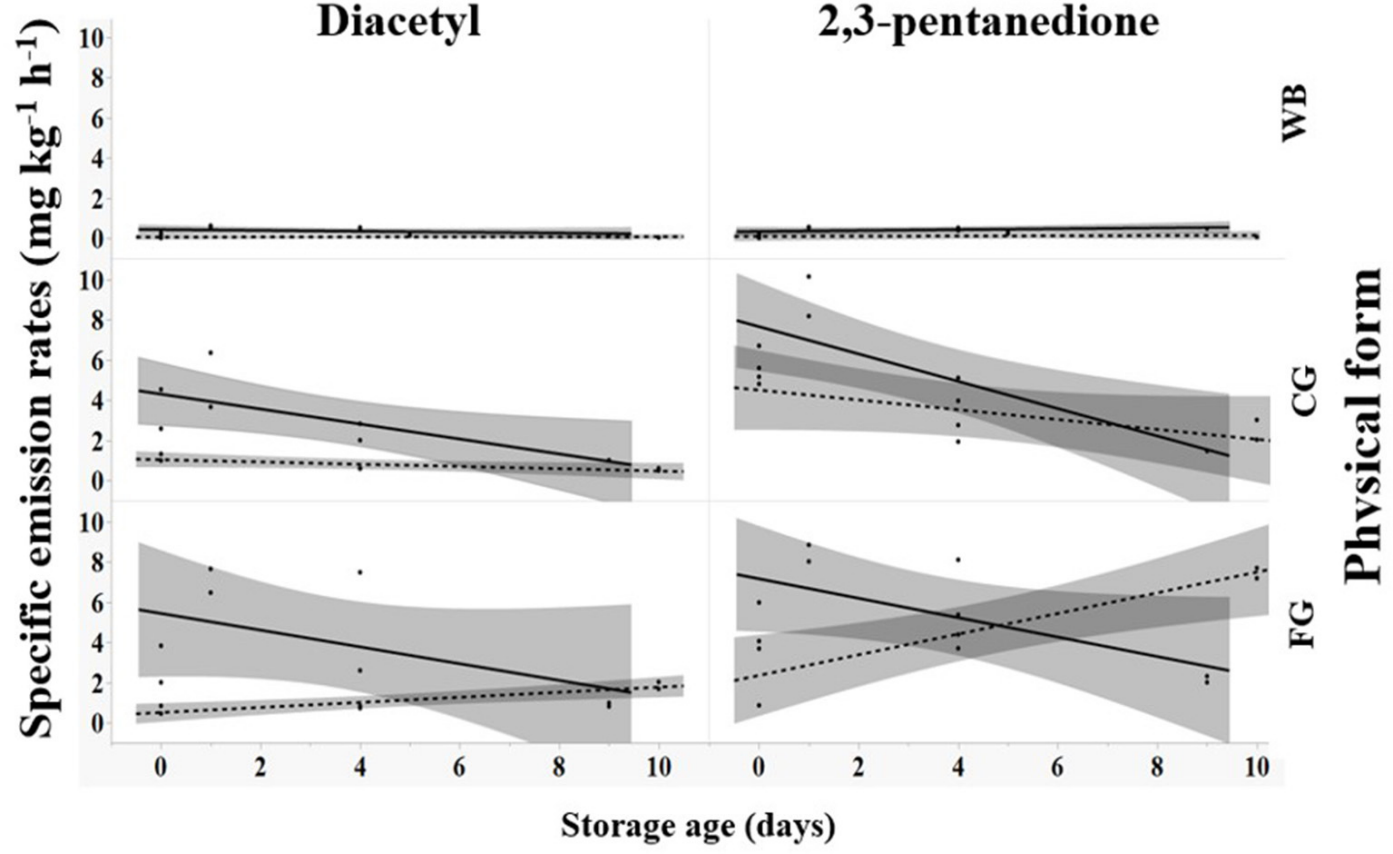
Effect of storage age (days) on specific emission rates for diacetyl and 2,3-pentanedione for different roast levels (— dark, - - - light) and forms of roasted coffee including whole bean (WB), coarse ground (CG), and fine ground (FG). Shaded areas around lines indicates 95% confidence limit of least-squares regression line.

When coffee was stored, SERs for diacetyl and 2,3-pentanedione decreased for dark roast (solid black line, Figure 2) in coarse ground or fine ground forms. SERs increased for light roast in fine ground forms but were unchanging for diacetyl and decreased for 2,3-pentanedione in coarse ground forms (dashed gray line, Figure 2; Supplementary Table S1). Dark roast, ground coffee exhibited an initial increase in emission factors on day 1 followed by a decrease. The highest average emission factors were observed on day 1, dark roast for both chemicals: fine ground for diacetyl (7.07 mg kg^−1^ h^−1^) and coarse ground for 2,3-pentanedione (9.17 mg kg^−1^ h^−1^). Whole bean emission factors were constant for light roast regardless of chemical and for dark roast for 2,3-pentanedione, but decreased for dark roast for diacetyl, never exceeding 0.64 mg kg^−1^ h^−1^ (Supplementary Table S1).

### Predicted Air Concentrations and Exposure Profile

#### Scenario A

**Figure 3:**
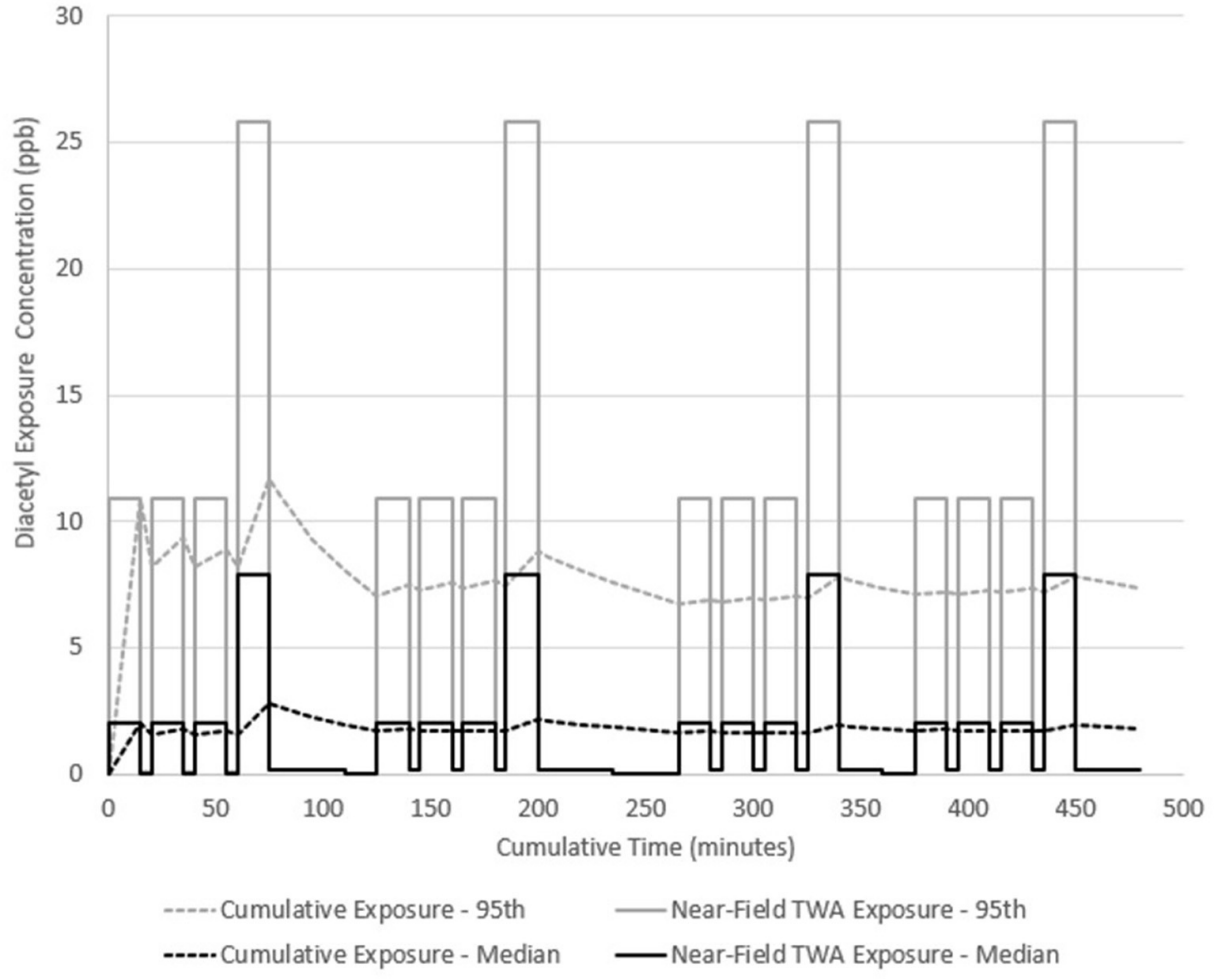
Diacetyl cumulative exposure profile for a general production worker (Scenario A). NIOSH REL 5.0 ppb for diacetyl.

Diacetyl emission rates for dark roast, whole bean and fine ground coffee were used to simulate a hypothetical employee exposure for an 8-h workday in a facility representative of one observed during field investigations. In scenario A, a general production worker performs three packaging tasks on whole bean coffee and one grinding task with this cycle of tasks repeated four times. Each task is performed on 10 kg of dark roast coffee (the source) for 15 min. Low exposures (0.04–0.21 ppb) were assumed to be the same concentration as far-field (away from source) exposure estimates in between tasks for 5 min, and during cleaning and labeling tasks for 15 min. Two 15-min breaks and one 30-min lunch are included with no exposure during these periods. The model estimated median near-field exposures at 2.1 ppb (95th percentile 10.9 ppb) during packaging and 7.9 ppb (95th percentile 25.8 ppb) during grinding. Because the 95th percentile encompasses the NIOSH short-term exposure limit (STEL) of 25 ppb, some grinding tasks could have exceeded the STEL. Total emitted mass of diacetyl was 0.53 mg during each packaging task and 9 mg during each grinding task. Diacetyl time-weighted average (TWA) cumulative exposure reached a maximum of 11.7 ppb after the first grinding task at 75 min and continued to increase and decrease throughout the day because of repeated exposures to diacetyl from roasted coffee sources with grinding having the greatest effect on cumulative exposure (Figure 3). By the end of the day, the full-shift cumulative exposure concentration was 1.8 ppb median (95th percentile 7.4 ppb) and might have exceeded the NIOSH recommended exposure limit (REL) of 5.0 ppb considering the confidence interval encompassed this limit.

#### Scenario B

**Figure 4:**
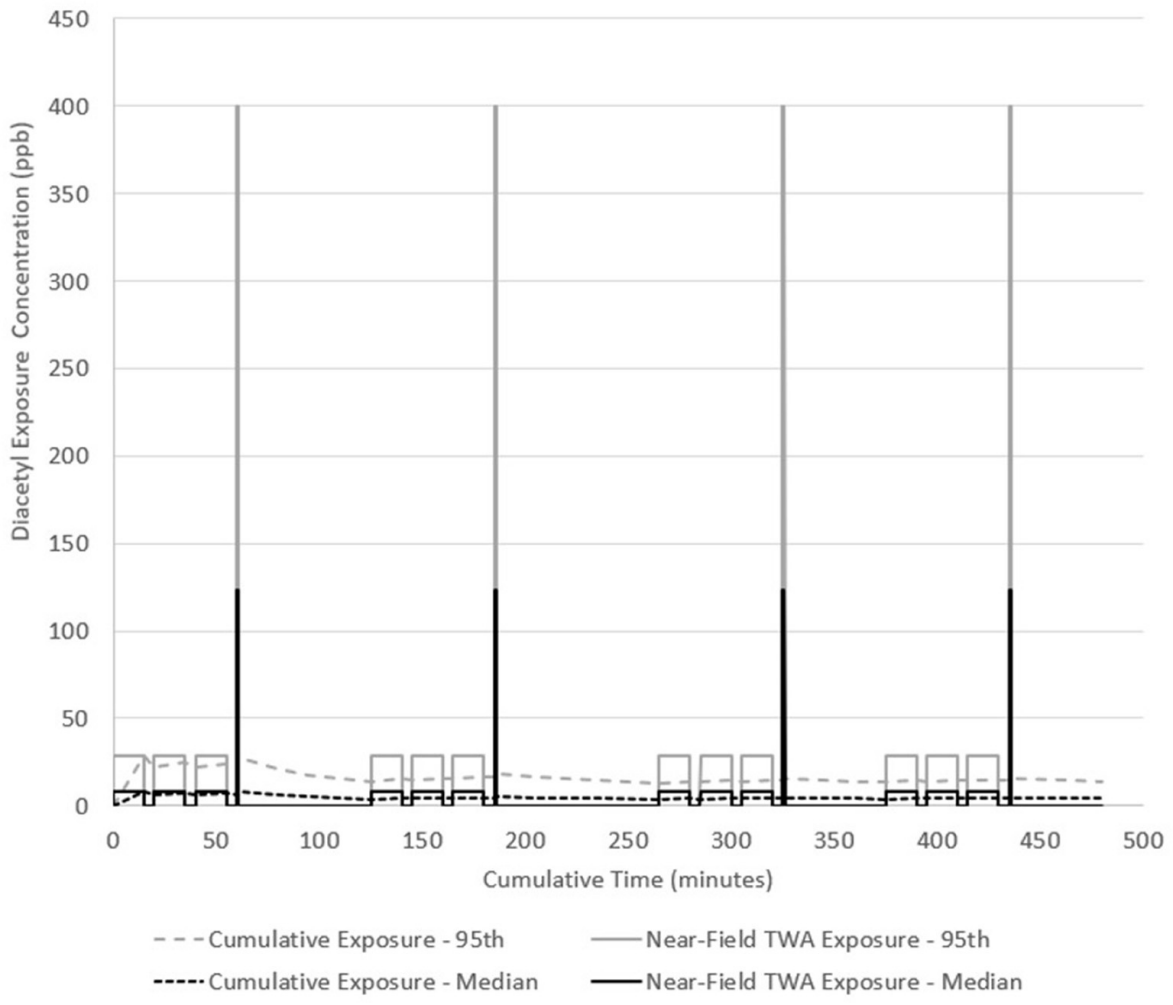
Diacetyl cumulative exposure profile for a general production worker (Scenario B). NIOSH REL 5.0 ppb for diacetyl.

The same work pattern occurs in scenario B but the grinding activity is assumed to last 1 min because of the quantity of coffee and large footprint of the grinder while the packaging task is 15 min. Packaging and grinding tasks were performed on 181.8 kg of dark roast coffee (the source). Low exposures (0.001–0.03 ppb) were used as far-field (away from source) exposure estimates in between tasks for 5 min, and during cleaning and labeling tasks for 15 min. Two 15-min breaks and one 30-min lunch are included with no exposure during these periods. The model estimated median near-field exposures at 8.3 ppb (95th percentile 28.9 ppb) during packaging for 15-min each and 123.5 ppb (95th percentile 28.9 ppb) during grinding for 1-min each. Total emitted mass of diacetyl was 9.6 mg during each packaging task and 11.4 mg during each grinding task. Assuming 14-min of far-field exposure to 0.2 ppb, grinding for 1-min would expose a worker to a cumulative 15-min TWA exposure of 8.4 ppb and would not exceed the NIOSH STEL of 25 ppb. Diacetyl TWA cumulative exposure was greatest at 8.3 ppb after the first packaging task (Figure 4). By the end of the day, the cumulative exposure concentration (i.e., full-shift TWA) was 4.1 ppb (95th percentile 14.2 ppb) and might have exceeded the NIOSH REL of 5.0 ppb considering the confidence interval encompassed this limit. Median short-term concentration excursions up to 4.9-times the STEL (123.5 ppb/25 ppb) occurred four times in this scenario.

### Sensitivity Analysis of Inter-zone Air Flow Rate (**β**)

**Figure 5:**
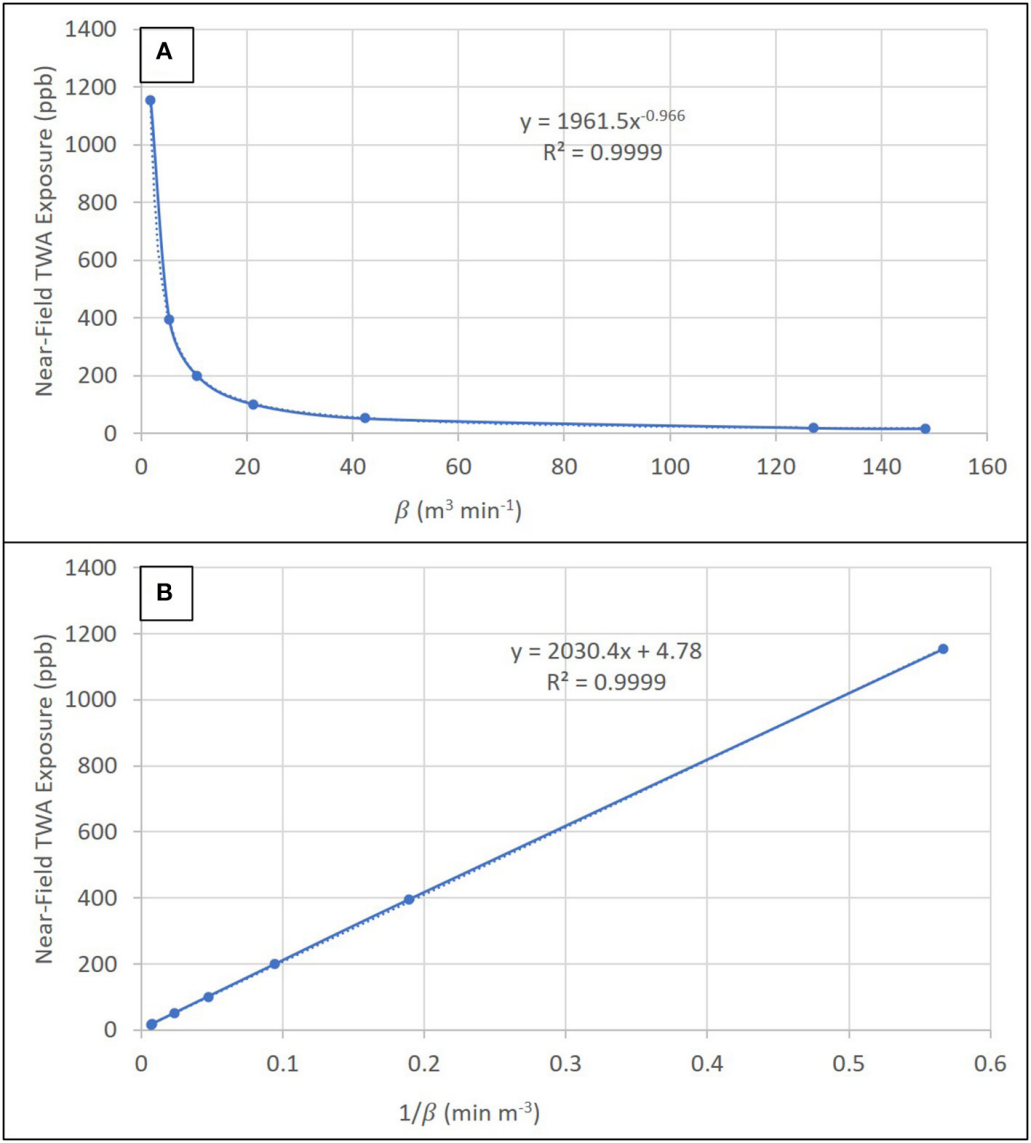
Sensitivity analysis showing the trend in near-field TWA exposure concentration as a function of **(A)** the inter-zone air flow rate (β) and **(B)** 1/β.

For 10 kg of fine ground, dark roast coffee, a sensitivity analysis of inter-zone air flow rate between near-field and far-field zones (β) demonstrated a substantial influence of low air flow rates, which are heavily influenced by random air velocities at the boundary (S), on near-field TWA exposure concentrations (Figure 4). Near-field TWA exposure concentrations decreased 2.9-fold (1,153–394 ppb) for β from 1.77 to 5.3 m^3^ min^−1^ in relatively still air corresponding to air velocities of 1.0–3.0 m min^−1^ and these concentrations asymptotically approached zero as β increased (Figure 5A). Near-field TWA exposure concentrations decreased 3.9-fold (200–51 ppb) for β from 10.6 to 42.4 m^3^ min^−1^ indicative of typical air velocities from 6.0 to 24 m min^−1^ in occupational settings. At air velocities indicative of walking (72–84 m min^−1^), near-field TWA exposure concentrations decreased 1.1-fold (18.4–16.0 ppb). As we approach steady-state conditions (i.e., t gets large), the near-field concentration can be approximated with a reduced form of Equation S1 (Supplementary Material) with no dependence on near-field or far-field volumes or air exchange rates in these fields. Plotting the inverse of β demonstrated a known linear trend from the reduced form of Equation S1 with the slope equivalent to the generation (2040) and intercept equivalent to generation divided by the flow rate (4.78) (Figure 5B).

## Discussion

Previous reports presented information regarding hazardous chemicals including diacetyl and 2,3-pentanedione and raised respiratory health concerns from exposure to workers at coffee roasting and packaging facilities. Scientific insight to the factors involved in emission of diacetyl and 2,3-pentanedione from roasted coffee, specifically roast level and physical form, and how these factors influence worker exposures has been lacking. Our study provides valuable SER values for these two chemicals from roasted coffee as well as a meaningful link between these emission factors and exposures through modeling based on information from actual workplaces observed during field investigations. The described approach investigates exposure air concentrations and SER depending on emission factors and coffee packaging and grinding tasks but also extends the application to real-world scenarios.

### Particle Size and Physical Form Effect on SERs

Although no statistical difference was observed between particle sizes of coarse and fine ground coffee, decreasing the particle size should increase the surface area available for emissions. We did observe a meaningful and statistical difference between chemical emission rates from these physical forms of roasted coffee (fine ground > coarse ground >> whole bean) for both chemicals. Emission rates from whole bean roasted coffee were the lowest because of the trapped gases in the bean pore structure and low surface area available for emissions compared to ground coffee. When the coffee was ground, these trapped gases were released and increased the emission rate for chemicals from the roasted coffee. Increased bean porosity for darker roasts (11) and the influence of this pore structure on emissions (25) likely affected estimates of SERs observed during freshly roasted and storage trials.

### Roast Level Effect on SERs

Unlike physical form, we observed a difference in the effect of roast level on chemical emissions that was dependent on the chemical. As the roast level darkened, SERs increased for diacetyl but decreased for 2,3-pentanedione. Schenker et al. observed increasing diacetyl and 2,3-pentanedione concentrations with increasing degrees of roast, with 2,3-pentanedione slightly higher than 2,3-butanedione (13). These researchers also observed 2,3-pentanedione decreasing at the darkest roast level which is similar to the observed drop in SERs observed in this study. Echt et al. also found that diacetyl emissions were the highest for the darkest roast level (French roast) and increased with increasing roast level (29).

### Storage Duration Effect on SERs

Longitudinal trends in emission factors during storage tests revealed a decrease for diacetyl and 2,3-pentanedione from dark roast level but an increase from light roast, especially for ground coffee. Emission factors for light roast would presumably decrease if the storage period was measured for a longer period. Pore structure differences between light and dark roast might have influenced emission factors of stored coffee. Increased bean porosity of darker roast may have increased the emission rates of chemicals decreasing the amount of chemical in the sample and decreasing the emission rates of chemicals over time (i.e., steeper slope in the trend line of emission rates vs. storage days). Lighter roast coffee likely had smaller pores decreasing the ability of trapped gases to migrate to the emission surface. Whole bean coffee had the slowest change in SERs over time because of the lack of available surface area for emission compared to ground coffee.

### Predicted Air Concentrations and Exposure Profile

Mass-balance models can be used to model air concentrations of chemicals by tracking mass through different zones or boxes. The well-mixed box model with a constant emission source is the simplest model to apply but assumes the chemical concentration is instantly dispersed throughout the space, which is not likely realistic. However, this simple model underestimates exposure at the source (30). Assessing the potential for occupational exposures to chemicals from an emission source can be more accurately approximated using a two-zone, near field/far field model, which accounts for incomplete mixing of air (31). Peak exposures at the emission source should be considered in epidemiologic and exposure studies because high concentration exposures for short durations can potentially overwhelm the body's natural defense mechanisms against adverse health effects (32). Near field/far field models are more realistic of these peak exposures when considering the employee may be near the emission source and exposed to higher concentrations, then move away from the emission source thus decreasing exposure. This approach still assumes complete and instantaneous mixing within each zone, only mass removal mechanism is through air leaving the zones, and input parameters that need to be measured or carefully assigned based on expert judgment or that require knowledge of the specific workplace scenario.

Model input parameters can be measured or estimated from other parameters but always have an uncertainty associated with them. Measured parameters can include room volume, ventilation rates, and emission rates, although the latter are often not measured and was an impetus to this work. Estimated parameters include inter-zone air flow rate, which can substantially affect near-field zone, and thus, worker exposure. A probabilistic approach to model input assigns an uncertainty bound to the parameter estimate and leads to a range of air concentrations that more accurately represents the variability observed in environmental sampling data (33). Distributions of input parameters are chosen to reflect what is known about the parameter. A uniform distribution, for example, might be chosen if the parameter has a range of values and the likelihood of any of the values is the same. A normal or lognormal distribution might be chosen if the parameter data fit these distributions, which have a measured or known variability (standard deviation or geometric standard deviation). Monte Carlo simulations are performed to sample these distributions multiple times (e.g., 10,000 iterations) and propagate the uncertainty in the input parameters to the model output.

Modeling worker exposures using SERs measured in the laboratory combined with observational data from the field and expert opinion yielded realistic exposure estimates that can be used to screen control strategies prior to implementation. We used a constant generation rate model, which is an appropriate choice given the limited change in emission rates over this time scale (15 min maximum). A modification to the model is available for situations when the emission source strength decreases over time (34). For this study, we only modeled two scenarios as examples of how to use the emission rates to estimate worker exposures. We also restricted the simulations to hypothetical non-flavoring coffee facilities with realistic model inputs based on observations during field surveys. For scenario A, we estimated median exposures to diacetyl of 2.1 ppb for packaging whole bean coffee and of 7.9 ppb for grinding coffee for intermittent short-term durations (15 min) leading to a full-shift exposure of 1.8 ppb for a general production worker performing various tasks and durations used in this simulation. Some exposures would likely exceed short-term STELs and full-shift RELs based on 95th percentile estimates encompassing these limits. For scenario B, we estimated median exposures to diacetyl of 8.3 ppb for packaging whole bean coffee (over 15 min) and of 123.5 ppb for grinding coffee (over 1 min) leading to a full-shift exposure of 4.1 ppb for a general production worker performing various tasks and durations used in this simulation. In this hypothetical scenario, multiple peak excursions above STELs for very short durations produced low estimates of full-shift exposures when averaged over the workday because of dilution from exposure to far-field exposures. Although this fixed cyclic pattern of peak exposures does not likely exist in occupational settings, episodic exposures to elevated concentrations of hazardous chemicals are likely in the coffee industry and depend on the work process and flow as well as individual worker behaviors and proximity of the worker to the source of emissions. Total mass emitted for the tasks in each scenario never exceeded 5% of the total predicted mass in the coffee based on 19 μg diacetyl/g coffee (15) indicating the model was producing reasonable estimates of emission for these chemicals. Modeled exposures presented in this study are similar to measured exposure estimates previously reported. Echt et al. observed median full-shift TWA exposures of 16 ppb diacetyl and 6.9 ppb 2,3-pentanedione among seven workers in a craft roastery (29). Davey et al. measured air concentrations of 0.02–8 ppb diacetyl and 2,3-pentanedione using sorbent tubes and peak excursions of these compounds between 15 and 20 ppb using proton-transfer reaction time-of-flight mass spectrometry (35). McCoy et al. measured two short-term breathing zone samples collected from a grinder operator in excess of 20 ppb (36). Pengelly et al. observed that 40% of full-shift personal samples exceeded 20 ppb but these samples originated from one worksite (37). In 17 coffee facilities, NIOSH researchers observed a comparable geometric mean of short-term task exposures of 8.6 ppb (GSD 2.8) for packaging coffee and 26 ppb (GSD 3.2) for grinding coffee (sampling duration ranged from 2 to 18 min) (1). At these non-flavoring coffee facilities, they estimated a geometric mean of full-shift exposures at 6.3 ppb (GSD 2.6).

### Sensitivity Analysis of Inter-zone Air Flow Rate (**β**)

Sensitivity analysis of inter-zone air flow rate demonstrated a substantial influence of this parameter on modeled worker exposures (i.e., near-field air concentrations). The most rapid change in near-field exposures occurred from 1.77 (relatively still air) to 10.6 m^3^ min^−1^ (air velocity 6 m min^−1^). The choice of air velocity at the boundary between zones is crucial to accurately represent real-world conditions. When the source is stationary (i.e., the air is still), chemical diffusion dominates the estimates for exposure. When air velocities increase because of movement of the source either from process (e.g., automation such as a conveyor belt) or worker movement, the air velocity at the boundary increases leading to worker exposures that can increase or decrease depending on work activities and the orientation and proximity of the worker to the source. Incorporating uncertainty in this input parameter is paramount to accurately reflect the variability observed in environmental measurements because of irregular worker activities (38). While not assessed here, we note that general ventilation values do not substantially affect near-field source concentration estimates, which are controlled by source emission characteristics (generation) and inter-zone air flow rate (removal).

Emission rates of diacetyl and 2,3-pentanedione and the results of modeled exposures support the need to control source emissions to control worker exposures. Our models indicate that the use of engineering controls such as local exhaust ventilation targeted at grinding machines could be beneficial to reduce exposures because we have shown that ground coffee releases these chemicals at a substantially greater rate than whole bean coffee. If engineering controls are not practicable in a workplace, modification of work practices to reduce the amount of time that the worker is near the source (roasted coffee) could be used to control exposure.

### Limitations

We observed an increase in SERs on day 1 for dark roast coffee but we did not measure SERs for day 1 storage on light roast coffee. We are uncertain whether an initial increase in SERs would be observed for light roast like that seen in dark roast. This would not substantially affect the trend observed where SERs increased with increasing storage age for light roast coffee. SERs were generated in this study to investigate the influence of roast level and grind. SERs reported here are limited to the test conditions and material tested. The assessment of particle size using Feret diameter did not accurately capture the decrease in particle size, which we hoped would act as a surrogate for the increase in surface area between coarse ground and fine ground forms of coffee. These results should not be generalized to workplace conditions but can be used to estimate air concentrations. Industrial coffee grinders used in coffee roasting and packaging facilities might grind roasted coffee to a different particle size than the grinder used in this study. Different particle sizes, and effective surface areas, will affect SERs. Future models could incorporate the age of the coffee and storage conditions to better represent the chemical emission rates. We present model scenarios to demonstrate the use of modeling to predict air concentrations and occupational exposures in this industry. Estimated air concentrations or exposures should be confirmed with air sampling. If broadly applicable SERs are desired, a wide range of coffee origins and species as well as roasting profiles should be assessed. SERs were experimentally derived at normal laboratory temperature and a fixed humidity. SERs increase with increasing environmental temperature for most compounds depending on vapor pressures and humidity for polar compounds, such as diacetyl in this study and formaldehyde. For example, a coffee roasting facility in hot, humid environment may have higher SERs than those in cold, dry environments.

## Conclusions

Chemicals including diacetyl and 2,3-pentanedione are emitted from roasted coffee at various rates depending on the roast level and physical form of the roasted coffee. SERs of diacetyl from freshly roasted coffee increased with roast level and grinding. SERs of 2,3-pentanedione did not change with roast level but increased with increasing level of grind. SERs of whole bean coffee remained stable in contrast to those of ground coffee, which decreased over a 10-day period. The exception to this was light roast coffee whose emission rates increased over a 10-day period. SERs developed here coupled with facility information obtained during previous field surveys provided model input to estimate worker exposures during various activities. Modeling demonstrated that near-field exposures depend on proximity to the source, duration of exposure, and air velocities in the near-field further supporting previously reported chemical air measurements in coffee roasting and packaging facilities.

## Data Availability Statement

The original contributions presented in the study are included in the article/Supplementary Material, further inquiries can be directed to the corresponding author/s.

## Author Contributions

RL conceived and designed the study, conducted the data analysis, and wrote the first draft of the manuscript. EF collected the samples. All authors read, reviewed, and approved the final manuscript.

## Funding

This work was supported by the National Institute for Occupational Safety and Health (NIOSH).

## Author Disclaimer

The findings and conclusions in this report are those of the authors and do not necessarily represent the official position of the National Institute for Occupational Safety and Health, Centers for Disease Control and Prevention. Mention of any company or product does not constitute endorsement by the U.S. Government, National Institute for Occupational Safety and Health, or Centers for Disease Control and Prevention.

## Conflict of Interest

The authors declare that the research was conducted in the absence of any commercial or financial relationships that could be construed as a potential conflict of interest.

## Publisher's Note

All claims expressed in this article are solely those of the authors and do not necessarily represent those of their affiliated organizations, or those of the publisher, the editors and the reviewers. Any product that may be evaluated in this article, or claim that may be made by its manufacturer, is not guaranteed or endorsed by the publisher.
