# Supplementary material for: Model Predictions of Occupational Exposures to Diacetyl and 2,3-Pentanedione Emitted From Roasted Whole Bean and Ground Coffee: Influence of Roast Level and Physical Form on Specific Emission Rates
Source: Front Public Health. 2022 Mar 23;10:786924. doi: 10.3389/fpubh.2022.786924 (PMC8983963; doi:10.3389/fpubh.2022.786924)
Supplement: Supplementary file 1 [file Data_Sheet_1.pdf]

# Supplementary Material
# Model predictions of occupational exposures to diacetyl and 2,3-pentanedione emitted from roasted whole bean and ground coffee: Influence of roast level and physical form on emission rates

**Ryan F. LeBouf*, Anand Ranpara, Elizabeth Fernandez, Dru A. Burns, Alyson R. Fortner**

Respiratory Health Division, National Institute for Occupational Safety and Health, Morgantown, WV USA

* Correspondence:
Ryan F. LeBouf
rlebouf@cdc.gov

The model to calculate near-field air concentrations is displayed in equation S1.

$$C_N(t) = \frac{G}{Q} + \frac{G}{\beta} + G\left(\frac{\beta \cdot Q + \lambda_2 \cdot V_N(\beta + Q)}{\beta \cdot Q \cdot V_N(\lambda_1 - \lambda_2)}\right)\exp(\lambda_1 \cdot t) - G\left(\frac{\beta \cdot Q + \lambda_1 \cdot V_N(\beta + Q)}{\beta \cdot Q \cdot V_N(\lambda_1 - \lambda_2)}\right)\exp(\lambda_2 \cdot t) \quad \text{(S1)}$$

The far-field model is displayed in equation S2.

$$C_F(t) = \frac{G}{Q} + G\left(\frac{\lambda_1 \cdot V_N + \beta}{\beta}\right)\left(\frac{\beta \cdot Q + \lambda_2 \cdot V_N(\beta + Q)}{\beta \cdot Q \cdot V_N(\lambda_1 - \lambda_2)}\right)\exp(\lambda_1 \cdot t) - G\left(\frac{\lambda_2 \cdot V_N + \beta}{\beta}\right)\left(\frac{\beta \cdot Q + \lambda_1 \cdot V_N(\beta + Q)}{\beta \cdot Q \cdot V_N(\lambda_1 - \lambda_2)}\right)\exp(\lambda_2 \cdot t) \quad \text{(S2)}$$

Where

$C_N$ = near-field concentration, mg m$^{-3}$

$C_F$ = far-field concentration, mg m$^{-3}$

G = generation rate, mg min$^{-1}$, calculated as the specific emission rate of the coffee in mg kg$^{-1}$ h$^{-1}$ multiplied by the mass of coffee in kg

Q = air ventilation rate, m$^3$ min$^{-1}$

$V_N$ = volume of the near-field, m$^3$, calculated from the near-field geometry, half-sphere with radius 0.75 m = 0.884 m$^3$. Value chosen to reasonably encompass a worker's breathing zone.

$V_F$ = volume of the far-field = total volume minus the $V_N$, m$^3$. Value used in calculation of $\lambda_1$.

$\beta = \frac{1}{2} \cdot FSA \cdot S$ = inter-zone air flow rate = 21.2 m$^3$ min$^{-1}$, where FSA=free surface area of near-field geometry of a half-sphere with radius 0.75 m = 3.53 m$^2$, and S = random air velocity at the boundary between near-field and far-field = 12 m min$^{-1}$ geometric mean (2 geometric standard deviation). Value chosen from freestream velocity representing

typical air velocities in occupational settings (Anthony and Anderson, 2013). Values chosen for sensitivity analysis include air velocities indicative of walking (Bohannon and Williams-Andrews, 2011).

$\lambda_1$ = air exchange rate in far field, min$^{-1}$, for equations see (Nicas, 2009). $|\lambda_1| \cong Q/V_F$

$\lambda_2$ = air exchange rate in near field, min$^{-1}$, for equations see (Nicas, 2009). $|\lambda_2| \cong \beta/V_N$

Supplementary Table S1. Average emission factors for diacetyl and 2,3-pentanedione at storage days after coffee roasting for different roast levels (light, dark) and physical forms including whole bean (WB), coarse ground (CG), and fine ground (FG).

| | | | | Emission Factors (mg kg$^{-1}$ h$^{-1}$) | | |
|---|---|---|---|---|---|---|
| Compound | Roast level | Physical form | Storage age in (days) | Average | Minimum | Maximum |
| Diacetyl | Dark | WB | 0 | 0.21 | 0.16 | 0.27 |
| | | | 1 | 0.60 | 0.56 | 0.64 |
| | | | 4 | 0.47 | 0.40 | 0.54 |
| | | | 9 | 0.17 | 0.17 | 0.18 |
| | | CG | 0 | 3.56 | 2.59 | 4.54 |
| | | | 1 | 5.01 | 3.67 | 6.36 |
| | | | 4 | 2.42 | 2.01 | 2.83 |
| | | | 9 | 1.01 | 1.01 | 1.01 |
| | | FG | 0 | 2.92 | 2.01 | 3.83 |
| | | | 1 | 7.07 | 6.48 | 7.66 |
| | | | 4 | 5.05 | 2.61 | 7.49 |
| | | | 9 | 0.90 | 0.82 | 0.99 |
| | Light | WB | 0 | 0.03 | 0.02 | 0.03 |
| | | | 5 | 0.16 | 0.15 | 0.16 |
| | | | 10 | 0.04 | 0.04 | 0.04 |
| | | CG | 0 | 1.16 | 1.00 | 1.33 |
| | | | 4 | 0.61 | 0.59 | 0.62 |
| | | | 10 | 0.56 | 0.51 | 0.62 |
| | | FG | 0 | 0.65 | 0.46 | 0.84 |

| | | | | | | |
|---|---|---|---|---|---|---|
| | | | 4 | 0.79 | 0.74 | 0.84 |
| | | | 10 | 1.86 | 1.69 | 2.03 |
| 2,3-pentanedione | Dark | WB | 0 | 0.17 | 0.13 | 0.22 |
| | | | 1 | 0.56 | 0.54 | 0.57 |
| | | | 4 | 0.46 | 0.40 | 0.53 |
| | | | 9 | 0.51 | 0.50 | 0.52 |
| | | CG | 0 | 5.94 | 5.17 | 6.71 |
| | | | 1 | 9.17 | 8.18 | 10.15 |
| | | | 4 | 4.54 | 3.97 | 5.11 |
| | | | 9 | 1.48 | 1.48 | 1.48 |
| | | FG | 0 | 5.03 | 4.07 | 5.99 |
| | | | 1 | 8.44 | 8.03 | 8.85 |
| | | | 4 | 6.25 | 4.39 | 8.11 |
| | | | 9 | 2.17 | 2.01 | 2.32 |
| | Light | WB | 0 | 0.03 | 0.02 | 0.03 |
| | | | 5 | 0.27 | 0.24 | 0.30 |
| | | | 10 | 0.08 | 0.07 | 0.08 |
| | | CG | 0 | 5.21 | 4.81 | 5.60 |
| | | | 4 | 2.35 | 1.94 | 2.76 |
| | | | 10 | 2.53 | 2.04 | 3.02 |
| | | FG | 0 | 2.28 | 0.87 | 3.69 |
| | | | 4 | 4.54 | 3.70 | 5.38 |
| | | | 10 | 7.44 | 7.19 | 7.70 |

## References


Anthony, T.R., and Anderson, K.R. (2013). Computational Fluid Dynamics Investigation of Human Aspiration in Low-Velocity Air: Orientation Effects on Mouth-Breathing Simulations. *The Annals of Occupational Hygiene* 57**,** 740-757.

Bohannon, R., and Williams-Andrews, A.R. (2011). Normal walking speed: a descriptive meta-analysis. *Physiotherapy* 97**,** 182-189.

Nicas, M. (2009). "The Near Field/Far Field (Two-Box) Model with a Constant Contaminant Emission Rate," in *Mathematical Models for Estimating Occupational Exposure to Chemicals,* eds. C.B. Keil, C.E. Simmons & T.R. Anthony. 2nd ed (Fairfax, VA: AIHA), 47-52.
